# More Parents With Intellectual Disabilities than we Thought: A Short Report from England

**DOI:** 10.1111/jar.70052

**Published:** 2025-06-16

**Authors:** Beth Tarleton, Katy Burch

**Affiliations:** ^1^ University of Bristol Bristol UK; ^2^ Oxford Brookes University Oxford UK

**Keywords:** adult services, child protection, England, parents with intellectual disabilities, parents with learning disabilities, social worker training

## Abstract

**Background:**

This paper recognises that there has been a long history of research into support for parents with intellectual disabilities in England and a helpful approach to integrating adults with intellectual disabilities in society called ʻValuing Peopleʼ. This focus has now faded.

**Method:**

The paper draws together findings from three recent English studies.

**Results:**

One third of the cases involving babies in the child protection system involved parents with diagnosed intellectual disability or a borderline or specific learning disability. The other two studies found that general adult social services were not set up to work with these parents, that social workers wanted more or better training and support and there was variable awareness of the Good Practice Guidance document.

**Conclusions:**

Parents with intellectual disabilities need to be on this Government's agenda; their need for tailored, on‐going support should also be acknowledged.

## 1 Introduction

This brief report discusses the current situation regarding support for parents with intellectual disabilities (known as learning disabilities) in England, drawing on 3 research projects whose findings were first presented in 2024 (Baginsky 2024; Tarleton et al. 2024; Burch et al. 2024). Taken together, these studies indicate that there are far more parents with an intellectual disability involved in care proceedings in England than previously believed and that the service response to these parents is patchy, even though there is good practice guidance (DoH/DfES 2007).

## 2 Contextual Information

There is a long history of research about these parents in England, including seminal work by Booth and Booth regarding numbers of parents with learning disabilities involved with the child protection system and how parents, particularly mothers, can be supported and advocate for themselves (Booth 2000, Booth and Booth 2003). Since then, there has been further research examining how parents are treated and good practice guidance detailing how they should be supported published in 2007 (DoH/DfES 2007). The guidance was championed by parents with learning disabilities and professional organisations at a time when the UK Government was also developing the 2001 Government White paper Valuing People: a new strategy for learning disability in the 21st Century.

Whilst the Good Practice Guidance was positively welcomed, and Wales and Scotland subsequently developed their own guidance (Scottish Consortium for Learning Disabilities (SCLD) 2015; Welsh Government 2023), the then UK Government's lack of commitment to parents with intellectual disabilities was signalled when it left it to the Working Together with Parents Network (WTPN) to disseminate the Guidance. This meant that local authorities were not supported or directed, by the Government, to integrate this non‐statutory guidance into local practice. The WTPN updated the Good Practice Guidance twice (WTPN 2016, 2021) and continued to promote it. The Guidance was eventually endorsed by two Presidents of the Family Division (Family Proceedings: Parents with a Learning Disability ‐ Courts and Tribunals Judiciary; Speech by the President of the Family Division: Parents with intellectual impairment in public law proceedings ‐ the need to be alert ‐ Courts and Tribunals Judiciary https://www.judiciary.uk/guidance‐and‐resources/family‐proceedings‐parents‐with‐a‐learning‐disability/ https://www.judiciary.uk/speech‐by‐the‐president‐of‐the‐family‐division‐parents‐with‐intellectual‐impairment‐in‐public‐law‐proceedings‐the‐need‐to‐be‐alert/) which meant that judges and other legal professionals could be made aware of it.

## 3 What Was the Situation in 2024?

The findings from the three 2024 studies are now presented in relation to two issues:

- Definitions and related understanding of the number of parents with intellectual (learning) disabilities or learning difficulties.
- The service response to parents.

### 3.1 Definitions and Numbers

In addition to parents with a diagnosed intellectual disability, it is widely recognised that there is a group of parents with mild or borderline intellectual disabilities who have similar support needs (Tarleton and Turney, 2019). There are no reliable English statistics but the number of parents with intellectual disabilities in the general population or within care proceedings has always been considered relatively small and the work undertaken to support them ‘niche’.

Burch et al.’s report demonstrated conclusively that the number of parents involved with care proceedings was far higher than previously recognised:

in 34% (67) of 200 recently concluded care proceedings regarding babies across four local authorities [in England], there was reliable, mostly expert, evidence that at least one parent had learning disabilities or learning difficulties …

These figures were broken down and included:

- Parents with an indicator of intellectual (learning) disabilities (overall IQ of below 70): 27 (45%).
- Parents with an indicator of borderline intellectual (learning) disabilities or learning difficulties: (overall IQ of between 70 and 85): 28 (47%).
- Parents with specific learning difficulties: 5 (8%).

Burch et al.'s findings suggest that (social) work with these parents should no longer be considered niche and call for improvements in both the tailoring of social work practice and the commissioning of support services specifically for this group of parents.

### 3.2 Service and System Response

Tarleton et al’s project found that adult social workers considered ‘learning disability teams’ (specific teams focused on working with adults with intellectual disabilities) to be perfectly placed to support parents with any level of intellectual disability. They had the knowledge and skills required. However, these teams usually only worked with parents with a diagnosed intellectual disability (in practice, when parents were assessed to have an overall IQ of below 70).

In all 3 studies, awareness of the Good Practice Guidance was reported to be very variable. Baginsky (2024) considered awareness to be really poor. The three studies suggest that, whilst there were examples of positive or even excellent practice, in line with the Good Practice Guidance, this was overall very patchy across England.

The studies also suggested a number of inter‐related issues inhibiting local authorities with social care responsibilities (adults and children's) from providing support to parents with intellectual disabilities ‐ particularly for parents with a milder impairment who were unable to access the ‘learning disability team’. These included:

- Social workers, from both Children's and Adults services, wanting to support parents but lacking training and confidence.
- In adult services:
  ○ Assessments of parents' support needs that were often very delayed.
  ○ A lack of clarity about or desire to provide support to parents with milder impairments under the Care Act (2014) which entitles disabled people to support.
- A lack of knowledge or understanding between adults' and children's social workers for example about each others role.
- Adults and children's service systems not working together effectively.
- A lack of advocacy services available to support parents during their involvement with children's social care (child protection) services or care proceedings, and a lack of support for parents if children are removed from their care.

## 4 Conclusion

The ability of local authorities to provide parents with support has been severely affected by austerity, funding cuts and previous UK governments' lack of political will to implement legislation or best practice guidance effectively. Parents with intellectual disabilities need to be on this UK Government’s agenda and their need for tailored, on‐going support should be acknowledged as something that is ‘normal’, just as all families need support (Spencer et al., 2024).

## Ethics Statement

The studies were approved by our respective universities and each contributing local authority's research governance process was followed.

## Consent

All of the participants provided fully informed consent which included permission to publish and archive their anonymised data.

## Conflicts of Interest

The authors declare no conflicts of interest.

## Data Availability

The data have been archived at our respective Universities.

## References

[jar70052-bib-0001] Baginsky, M. 2024. Hidden in Plain Sight: Parents With a Learning Disability in Childcare Proceedings and Beyond. NIHR, Kings College.

[jar70052-bib-0002] Booth, T. 2000. “Parents With Learning Difficulties, Child Protection and the Courts.” Representing Children 13, no. 3: 175–188.

[jar70052-bib-0003] Booth, T. , and W. Booth . 2003. “Self‐Advocacy and Supported Learning for Mothers With Learning Difficulties.” Journal of Learning Disabilities 7, no. 2: 165–193.

[jar70052-bib-0004] Burch, K. , A. Simpson , V. Taylor , A. Bala , and S. Morgado De Queiroz . 2024. “Babies in Care Proceedings: What Do We Know About Parents With Learning Disabilities or Difficulties?” Nuffield Family Justice Observatory. https://www.nuffieldfjo.org.uk/resource/babies‐in‐care‐proceedings‐what‐do‐we‐know‐about‐parents‐with‐learning‐disabilities‐or‐difficulties.

[jar70052-bib-0005] DoH/DfES . 2007. Good Practice Guidance on Working With Parents With a Learning Disability [ARCHIVED CONTENT]. Department of Health ‐ Publications and Statistics.

[jar70052-bib-0006] Scottish Consortium for Learning Disabilities (SCLD) . 2015. “Supported Parenting: Refreshed Scottish Good Practice Guidelines for Supporting Parents With a Learning Disability.” SCLF. Supported_Parenting_web.pdf (scld.org.uk).

[jar70052-bib-0007] Spencer, M. , B. Tarleton , S. Collings , G. McIntyre , and D. Turney . 2024. “If We Know What Works, Why Aren't We Doing It?” British Journal of Social Work 54, no. 6: 2808–2825. 10.1093/bjsw/bcae080.

[jar70052-bib-0011] Tarleton, B. , and D. Turney . 2019. “Understanding 'Successful Practice(s)' with Parents with Learning Difficulties when there are Concerns about Child Neglect: The Contribution of Social Practice Theory.” Child Indicators Research 13: 387–409. https://link.springer.com/article/10.1007/s12187‐019‐09682‐y.

[jar70052-bib-0008] Tarleton, B. , G. MacIntyre , and R. J. Fawcett . 2024. How Do Adult and Learning Disability Social Workers Engage With Parents With Learning Disabilities? University of Bristol. https://researchinformation.bris.ac.uk/ws/portalfiles/portal/422858967/Summary_Adult_services_and_parents_with_learning_disabilities_1_.pdf.

[jar70052-bib-0009] Welsh Government . 2023. “Guidance for Social Workers for Families Where the Parent has a Learning Disability.” https://www.gov.wales/guidance‐social‐workers‐families‐where‐parent‐has‐learning‐disability‐html.

[jar70052-bib-0110] WTPN . 2016. Working Together With Parents Network (WTPN) Further Update of the DoH/DfES Good Practice Guidance on Working With Parents With a Learning Disability. University of Bristol. FINAL 2016 WTPN UPDATE OF THE GPG.pdf (bristol.ac.uk).

[jar70052-bib-0010] WTPN . 2021. Working Together With Parents Network (WTPN) Further Update of the DoH/DfES Good Practice Guidance on Working With Parents With a Learning Disability. University of Bristol. FINAL 2021 WTPN UPDATE OF THE GPG.pdf (bristol.ac.uk).

